# Mapping the Knowledge Landscape of Acupuncture for Primary Headaches: A Bibliometric Analysis From 2005 to 2025

**DOI:** 10.1155/prm/4922234

**Published:** 2026-07-31

**Authors:** Gui-yu Liu, Jia-yong-ming Liu, Han-na Jin, Zi-yan Chen, Xiao-han Zhu, Wen-jing Tong, Zi-meng Lai, Jia-geng Xie, Qing-yu Chang, Wen-lin Yu, Wei Xie

**Affiliations:** ^1^ School of Traditional Chinese Medicine, Southern Medical University, Guangzhou, Guangdong, China, fimmu.com; ^2^ Department of Neurology, Huizhou Hospital of Guangzhou University of Chinese Medicine (Huizhou Hospital of Traditional Chinese Medicine), Huizhou, Guangdong, China; ^3^ Department of Traditional Chinese Medicine, Nanfang Hospital, Southern Medical University, Guangzhou, Guangdong, China, fimmu.com

**Keywords:** acupuncture, bibliometric analysis, migraine, primary headache, tension-type headache

## Abstract

**Objective:**

To provide an updated bibliometric overview of the research landscape, hotspots, and emerging trends of acupuncture for primary headaches (PH).

**Methods:**

Publications related to acupuncture for PH were retrieved from the Web of Science Core Collection (WoSCC) for the period from January 1, 2005, to December 31, 2025. Only English‐language articles and reviews were included. After manual screening based on title, abstract, publication year, document type, language, and topic relevance, 286 eligible publications were included. Bibliometric analyses and visualizations were performed using CiteSpace, VOSviewer, Bibliometrix, and Microsoft Excel.

**Results:**

The annual number of publications showed an overall upward trend, particularly after 2018. China was the leading contributor in terms of publication output and collaborative activity, followed by Germany, Italy, and the United States. Collaboration networks were identifiable at the country, institution, and author levels, although they remained concentrated among a limited number of leading countries and core research groups. The intellectual base of the field was mainly shaped by randomized controlled trials, systematic reviews, and evidence syntheses. Thematic analyses indicated that research was primarily centered on migraine, prophylaxis, efficacy, and headache‐related clinical outcomes. Tension‐type headache was also represented, whereas cluster headache and other less‐studied PH subtypes received comparatively limited attention. Mechanism‐related themes, including functional connectivity, neuroimaging, and trigeminocervical mechanisms, have emerged in recent years but remain less developed than the clinical literature.

**Conclusion:**

Research on acupuncture for PH has become increasingly productive, structured, and clinically evidence‐oriented over the past 2 decades. However, the field remains characterized by thematic concentration, selective collaboration, and relatively limited mechanistic depth. Future studies should place greater emphasis on underrepresented PH subtypes, broader interdisciplinary collaboration, and more rigorous translational and mechanistic investigation.

## 1. Introduction

Primary headaches (PH) are among the most common and disabling neurological disorders worldwide and include migraine, tension‐type headache (TTH), cluster headache, and several other PH syndromes [1]. These conditions impose a substantial burden on quality of life, daily functioning, work productivity, and healthcare utilization [2]. Among them, migraine has been particularly emphasized in both clinical practice and research because of its high prevalence, recurrent course, and strong association with disability [3], while TTH also contributes greatly to the overall public health burden [4]. Although pharmacological treatments remain central to headache management, limitations such as incomplete response, recurrence, medication overuse, adverse effects, and poor long‐term adherence have encouraged increasing interest in nonpharmacological and complementary treatment strategies [5].

Acupuncture has been widely used in the prevention and management of PH, especially migraine and TTH, and has attracted growing attention in both clinical practice and research [6, 7]. Over the past 2 decades, randomized controlled trials, systematic reviews, and evidence syntheses have suggested that acupuncture may provide benefit for headache frequency, pain intensity, and quality‐of‐life‐related outcomes in selected patient populations [6, 8–10]. At the same time, research in this field has expanded beyond efficacy evaluation alone to include comparative effectiveness, electroacupuncture, and, more recently, neuroimaging and mechanism‐related exploration [11–13]. As a result, the literature on acupuncture for PH has become increasingly extensive, multidisciplinary, and methodologically diverse.

Bibliometric analysis is a useful tool for mapping the development of a research field by identifying publication trends, influential contributors, collaboration networks, intellectual bases, and evolving research themes [14]. Several bibliometric studies have already explored acupuncture‐related research on headache, migraine, or TTH [15–17]. These studies have provided valuable insights into publication growth, major countries and institutions, and broad research hotspots. However, the existing literature still shows several limitations. First, many prior analyses have focused on headache in a broad sense or on migraine specifically, rather than on other PH as a more explicit conceptual framework. Second, the thematic distinction among major PH subtypes has not always been sufficiently clarified, which may obscure the current imbalance of research attention across migraine, TTH, cluster headache, and other subtypes. Third, earlier studies often placed greater emphasis on descriptive productivity indicators than on the combined interpretation of collaboration structures, intellectual‐base evolution, and thematic transitions. Finally, because the literature in this area overlaps with broader acupuncture and pain research, stricter topic‐based screening is needed to improve the specificity of bibliometric mapping.

Against this background, an updated and more tightly screened bibliometric evaluation of acupuncture research on PH is warranted. Such an analysis may not only describe how the field has grown, but also clarify how knowledge production is distributed across countries, institutions, and authors; which journals and references have shaped the field; and how research themes have shifted over time. Importantly, this approach may also help reveal structural features of the current literature, including the predominance of migraine‐related research, the relative underrepresentation of cluster headache and other less‐studied PH subtypes, and the continued dominance of clinical efficacy‐oriented studies over mechanistic and translational investigation.

Therefore, the present study conducted a bibliometric analysis of publications on acupuncture for PH indexed in the Web of Science Core Collection (WoSCC) from 2005 to 2025. By integrating publication trends, collaboration networks, cited journal dynamics, co‐cited reference analysis, thematic mapping, and trend‐topic analysis, this study aimed to provide an updated overview of the field, identify its major hotspots and intellectual structure, and highlight current gaps and future research directions.

## 2. Materials and Methods

### 2.1. Data Source and Search Strategy

The WoSCC was selected as the data source for this bibliometric analysis. Relevant publications were searched from January 1, 2005, to December 31, 2025, and the retrieval was performed on March 9, 2026. Only English‐language articles and reviews were included. The search strategy combined two thematic modules: acupuncture‐related interventions and PH disorders. The final search formula was constructed as follows: TS=(acupuncture OR electroacupuncture OR “manual acupuncture” OR “auricular acupuncture” OR “scalp acupuncture” OR “warming acupuncture” OR “fire needling” OR acupoint OR needling) AND TS=(“primary headache” OR “primary headache disorder∗” OR migraine∗ OR “tension‐type headache∗” OR “tension type headache∗” OR “cluster headache∗” OR “trigeminal autonomic cephalalgia∗” OR TACs).

### 2.2. Eligibility Criteria and Screening

Publications were included if they focused on acupuncture‐related interventions for the treatment or management of PH disorders, including migraine, TTH, cluster headache, and other trigeminal autonomic cephalalgias where applicable. Only articles and reviews published in English were retained. Publications were excluded if they concerned secondary headaches, if headache was only a minor symptom or comorbidity, if acupuncture was not the primary intervention of interest, or if the main topic was another pain condition rather than PH. Editorials, letters, meeting abstracts, corrections, and other nonarticle/nonreview document types were also excluded.

After retrieval, all records were exported with full records and cited references. Duplicate records were removed first. Then, two investigators independently screened titles and abstracts for relevance. For records with uncertain eligibility, full texts were further checked. Any disagreements were resolved through discussion with a third reviewer. Because the search strategy intentionally included broad acupuncture‐ and headache‐related terms, manual relevance screening was essential to exclude records in which headache was only a secondary symptom or acupuncture was not the primary intervention. The final eligible publications were included in the bibliometric analysis.

### 2.3. Literature Retrieval and Screening

**FIGURE 1:**
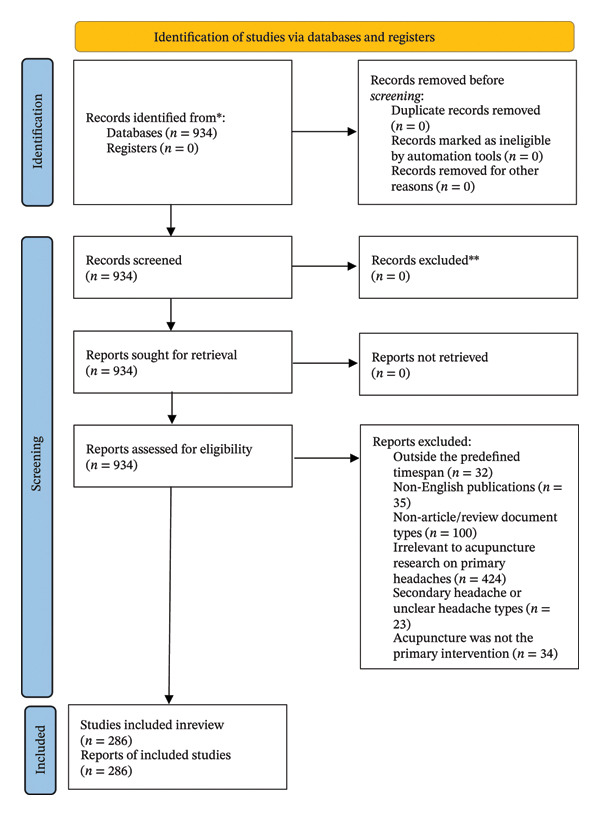
Flowchart of literature retrieval and screening. A total of 934 records were identified through WoSCC database searching. After manual screening based on title, abstract, publication year, language, document type, and topic relevance, 286 publications were finally included in the bibliometric analysis.

The literature retrieval and screening process is shown in Figure 1. A total of 934 records were identified through WoSCC database searching, and no duplicate records were found. All retrieved records were subsequently screened manually according to title, abstract, publication year, language, document type, and topic relevance. After screening, 648 records were excluded for the following reasons: outside the predefined timespan (*n* = 32), non‐English publications (*n* = 35), nonarticle/review document types (*n* = 100), records irrelevant to acupuncture research on PH (*n* = 424), studies on secondary headache or unclear headache types (*n* = 23), and studies in which acupuncture was not the primary intervention (*n* = 34). Ultimately, 286 eligible publications were included in the bibliometric analysis.

## 3. Results

### 3.1. Annual Publication Trend and Citation Pattern

**FIGURE 2:**
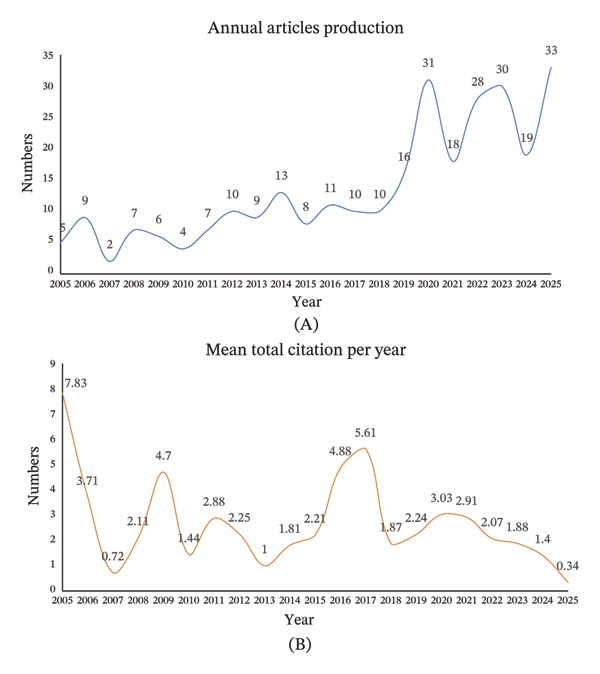
Annual publication output and average citations per publication from 2005 to 2025. (A) Annual number of publications on acupuncture for primary headaches. (B) Average citations per publication by publication year. Citation‐based values in recent years were relatively lower because newly published studies had less time to accumulate citations.

Figure 2 shows the annual publication trend and citation pattern of studies on acupuncture for PH from 2005 to 2025. As shown in Figure 2A, the annual number of publications displayed a fluctuating upward trend over the study period. During the early stage, annual output remained relatively low, generally ranging from 2 to 13 publications between 2005 and 2012. After 2018, publication output increased more noticeably, reaching 16 in 2019 and continuing to rise thereafter. The highest annual output was recorded in 2025, with 33 publications.

Figure 2B presents the average citations per publication by publication year. The highest value was observed in 2005 (7.83), followed by fluctuations in subsequent years, with another relative increase in 2016‐2017. Citation‐based values were lower in recent years, which are most likely attributable to the shorter time available for newly published studies to accumulate citations, rather than necessarily indicating lower quality or influence.

### 3.2. Country‐Level Contributions and Collaboration

A total of 47 countries contributed to research on acupuncture for PH. The top 10 most productive countries are listed in Table 1. China ranked first with 178 publications, accounting for 62.2% of the total literature included in this study. Germany ranked second with 20 publications, followed by Italy (*n* = 17), the United States (*n* = 12), and South Korea (*n* = 10). Publication output in most other countries was below 10, suggesting that research in this field remains concentrated in a limited number of countries. A further cumulative number of these 10 most productive countries is available in Figure S1A.

**TABLE 1:** Top 10 most productive countries in acupuncture research on primary headaches.

Rank	Country	Frequent	Total citation	Average article citations
1	China	178	3,147	17.7
2	Germany	20	2,096	104.8
3	Italy	17	317	18.6
4	USA	12	266	22.2
5	Korea	10	115	11.5
6	Spain	6	145	24.2
6	Turkey	6	60	10.0
8	Australia	5	184	36.8
8	Brazil	5	193	38.6
10	Iran	4	84	21.0

**FIGURE 3:**
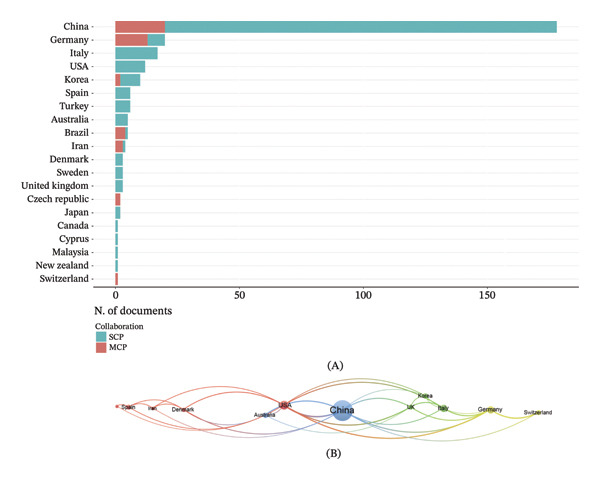
Country‐level contributions and collaboration patterns in acupuncture research on primary headaches. (A) Distribution of single‐country publications (SCP) and multiple‐country publications (MCP) among the leading countries. (B) Collaboration network among countries. Node size reflects publication output, and connecting lines indicate collaborative links. (C) Global map of country‐level collaboration. Darker shading indicates higher publication output, and thicker lines represent stronger collaborative ties.

The corresponding authors’ countries are shown in Figure 3A. China was the most productive country in this field, contributing substantially more publications than any other country, followed by Germany, Italy, the United States, South Korea, and Spain. Most countries showed a predominance of single‐country publications (SCP) over multiple‐country publications (MCP), indicating that domestic output remained the major mode of publication.

The country collaboration network is presented in Figure 3B. International cooperation was evident, but it was mainly centered on a limited number of high‐output countries, particularly China and the United States, which maintained collaborative links with Germany, Italy, the United Kingdom, Switzerland, and several other countries. Overall, the field has developed a recognizable international collaboration network, although cross‐national cooperation remains concentrated among a relatively small group of leading countries. Further international collaboration map is available in Figure S1B.

### 3.3. Institutional Contributions and Collaboration

A total of 793 institutions participated in research on acupuncture for PH. The top 10 most productive institutions are listed in Table 2. Chengdu University of Traditional Chinese Medicine ranked first with 98 publications, accounting for approximately one‐third of the total output in this field. It was followed by Beijing University of Chinese Medicine (*n* = 43) and Capital Medical University. Most of the leading institutions were located in China, whereas a smaller number of highly productive institutions were based in Germany, Italy, and the United States, including the University of Turin, Technical University of Munich, and Harvard University. Further cumulative number of these 10 most productive institutions is available in Figure S2A.

**TABLE 2:** Top 10 most productive institutions in acupuncture research on primary headaches.

Rank	Institution	Country	Article
1	Chengdu University Of Traditional Chinese Medicine	China	98
2	Beijing University Of Chinese Medicine	China	43
3	Capital Medical University	China	36
4	China Academy Of Chinese Medical Sciences	China	27
5	Zhejiang Chinese Medical University	China	23
6	China Medical University Taiwan	China	21
6	University of Turin	Italy	21
8	Technical University of Munich	Germany	19
9	Harvard University	USA	18
10	Shanghai University of Traditional Chinese Medicine	China	16

**FIGURE 4:**
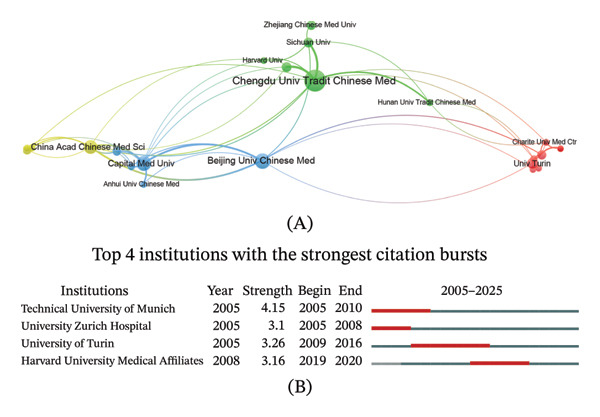
Institutional collaboration and citation burst analysis in acupuncture research on primary headaches. (A) Collaboration network among institutions. (B) Top institutions with the strongest citation bursts from 2005 to 2025.

The institutional collaboration network is shown in Figure 4A. Several Chinese institutions occupied central positions in the network, particularly Chengdu University of Traditional Chinese Medicine, Beijing University of Chinese Medicine, China Academy of Chinese Medical Sciences, and Capital Medical University. These institutions were closely connected with one another and also maintained collaborative links with several international institutions, including Harvard University, Charité University Medical Center, and the University of Turin. The time overlay network is available in Figure S2A.

Figure 4B presents the institutional citation burst analysis. The institutions with the strongest burst strength were the Technical University of Munich, University Zurich Hospital, University of Turin, and Harvard University Medical Affiliates. Notably, burst institutions were mainly concentrated in Europe and North America during earlier periods, whereas the current collaboration network was more strongly centered on Chinese institutions.

### 3.4. Author Collaboration and Productivity

A total of 3,218 authors contributed to research on acupuncture for PH. The top 10 most productive authors are listed in Table 3. Liang Fanrong was the most prolific author, with 30 publications and 991 citations, followed by Liu Lu (24 publications, 379 citations) and Zhao Ling (21 publications, 677 citations). Most of the leading authors were affiliated with Chengdu University of Traditional Chinese Medicine or Capital Medical University, highlighting the important roles of these institutions in this field. Further analysis of the production of these authors by Lotka’s Law is available in Figure S3A.

**TABLE 3:** Top 10 most productive authors in acupuncture research on primary headaches.

Rank	Name	Institutions	Articles	Articles fractionalized	Citation	h‐index
1	Liang, Fanrong	Chengdu University Of Traditional Chinese Medicine	30	2.9	991	14
2	Liu, Lu	Capital Medical University	24	2.47	379	13
3	Zhao, Ling	Chengdu University Of Traditional Chinese Medicine	21	2.14	677	9
4	Wang, Linpeng	Capital Medical University	20	2.23	453	8
5	Li Ying	Capital Medical University	17	1.7	960	13
6	Zheng, Hui	Chengdu University Of Traditional Chinese Medicine	17	2.22	673	12
7	Li, Zhengjie	Chengdu University Of Traditional Chinese Medicine	16	1.35	312	5
8	Allais, Gianni	University of Turin	15	2.19	868	10
9	Li Bin	University of Turin	13	1.25	200	10
10	Sun, Mingsheng	Chengdu University Of Traditional Chinese Medicine	13	1.42	122	6

**FIGURE 5:**
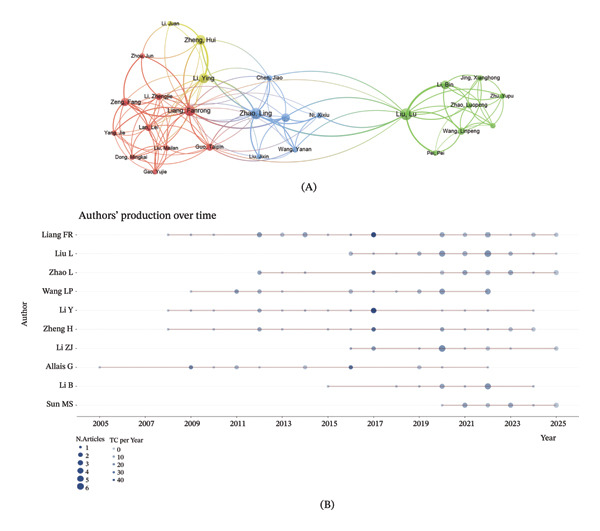
Author collaboration and publication patterns in acupuncture research on primary headaches. (A) Author collaboration network. Node size reflects the relative productivity of authors, and links indicate co‐authorship relationships. (B) Annual publication patterns of the leading authors. Bubble size indicates the number of articles, and color intensity reflects citations per year.

The author collaboration network is presented in Figure 5A. Several relatively stable collaborative clusters were identified. A large cluster centered on Liang Fanrong was closely connected with authors such as Zeng Fang, Li Ying, and Zheng Hui, whereas Zhao Ling occupied an intermediate bridging position linking multiple subgroups. Another distinct collaborative cluster was organized around Liu Lu and Li Bin. These findings suggest that the field has formed recognizable author communities, although collaboration remains concentrated within a limited number of core research groups. Further analysis of these authors in a specific topic is available in Figure S3B.

Figure 5B shows the temporal publication patterns of the leading authors. Several authors maintained sustained productivity across multiple years, while some authors, including Liu Lu, Li Bin, and Sun Mingsheng, became more active in the later years of the study period.

### 3.5. Source Distribution and Cited Journal Dynamics

A total of 251 journals published articles or reviews related to acupuncture for PH. The top 10 most productive journals are listed in Table 4. *Journal of Pain Research* published the largest number of articles (*n* = 23), followed by *Frontiers in Neurology* (*n* = 22). These were the only two journals that published more than 20 articles in this field. Other relatively active journals included *Frontiers in Neuroscience* (*n* = 12) and *Medicine* (*n* = 12). Overall, the most productive journals were mainly concentrated in the fields of neurology, pain research, and complementary medicine. Further analysis of these core journals is available in Figure S4.

**TABLE 4:** Top 10 most productive journals in acupuncture research on primary headaches.

Rank	Journal	Articles	Citations	Average citation	JCR division	IF	h‐index
1	Journal of Pain Research	23	176	7.65	Q2	2.5	8
1	Frontiers in Neurology	22	219	9.95	Q2	2.8	9
3	Frontiers in Neuroscience	12	177	14.75	Q2	3.2	9
3	Medicine	12	89	7.42	Q2	1.4	4
5	Cephalalgia	10	448	44.8	Q1	4.6	9
5	Neurological Sciences	10	169	16.9	Q3	2.4	8
7	Acupuncture in Medicine	9	183	20.33	Q2	2.6	7
7	BMJ Open	9	57	6.33	Q2	2.3	5
7	Evidence‐Based Complementary and Alternative Medicine	9	166	18.44	Q3	2.65	8
7	Trials	9	75	8.33	Q3	2	5

**FIGURE 6:**
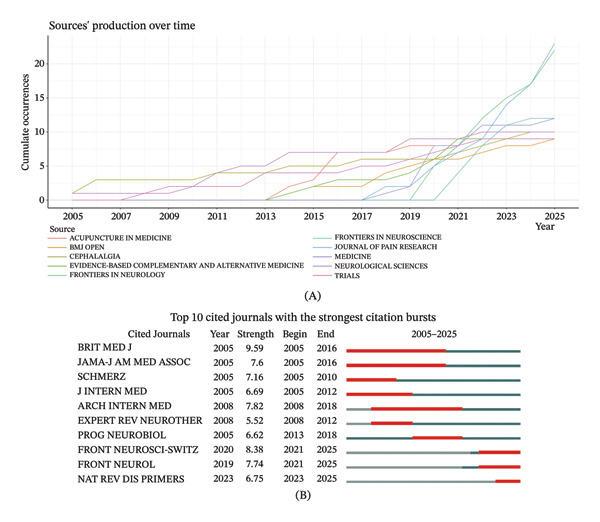
Source dynamics and cited journal burst analysis in acupuncture research on primary headaches. (A) Cumulative publication trends of the major sources from 2005 to 2025. (B) Top 10 cited journals with the strongest citation bursts from 2005 to 2025.

Figure 6A shows the cumulative production trends of the major sources. Journals such as *Frontiers in Neurology*, *Frontiers in Neuroscience*, *Medicine*, *Journal of Pain Research*, *Cephalalgia*, and *Trials* accounted for an increasing proportion of publications over time.

Figure 6B presents the citation burst analysis of cited journals. Early bursts were mainly observed in high‐impact general medical and clinical journals, such as *BMJ*, *JAMA*, *Journal of Internal Medicine*, and *Archives of Internal Medicine*, as well as review‐oriented journals including *Expert Review of Neurotherapeutics* and *Progress in Neurobiology*. In contrast, more recent bursts were concentrated in specialty journals such as *Frontiers in Neuroscience*, *Frontiers in Neurology*, and *Nature Reviews Disease Primers*. These findings indicate that the knowledge base of acupuncture research on PH has gradually become more specialized and increasingly connected with neurology and neuroscience.

### 3.6. Intellectual Base and Influential References

The 10 most highly cited publications in the field of acupuncture research on PH are presented in Table 5, and additional details are provided in Table S1. The most highly cited article was “Acupuncture for patients with migraine: a randomized controlled trial” by Linde K et al., with 465 citations [18]. This was followed by “Efficacy of acupuncture for the prophylaxis of migraine: a multicenter randomized controlled clinical trial” by Diener HC et al. (298 citations) [19] and “The Long‐Term Effect of Acupuncture for Migraine Prophylaxis: A Randomized Clinical Trial “by Zhao L et al. (294 citations) [20]. Notably, although Linde Klaus was not among the top 10 most productive authors, this author contributed four publications to the list of the most highly cited studies, indicating a prominent influence on the intellectual base of the field.

**TABLE 5:** Top 10 most highly cited publications in acupuncture research on primary headaches.

Rank	Paper	Doi	Total citations	TC per year	Normalized TC
1	Linde K, 2005, JAMA‐J Am Med Assoc	10.1001/jama.293.17.2118	468	51.79	2.72
2	Diener HC, 2006, Lancet Neurol	10.1016/S1474‐4422 (06)70382‐9	298	14.19	3.82
3	Zhao L, 2017, JAMA Intern Med	10.1001/jamainternmed.2016.9378	294	29.4	5.24
4	Melchart D, 2005, BMJ‐BRIT Med J	10.1136/bmj.38512.405440.8F	277	12.59	1.61
5	Linde K, 2009, Cochrane DB Syst Rev‐a	10.1002/14651858.CD001218.pub2	262	14.56	3.09
6	Linde K, 2016, Cochrane DB Syst Rev‐a	10.1002/14651858.CD001218.pub3	224	20.36	4.17
7	Xu SB, 2020, BMJ‐BRIT Med J	10.1136/bmj.m697	172	24.57	8.1
8	Li Y, 2012, CAN Med Assoc J	10.1503/cmaj.110551	135	9	3.99
9	Linde K, 2009, Cochrane DB Syst Rev	10.1002/14651858.CD007587	131	7.28	1.55
10	Li ZJ, 2016, Sci Rep‐UK	10.1038/srep20298	110	10	2.05

**FIGURE 7:**
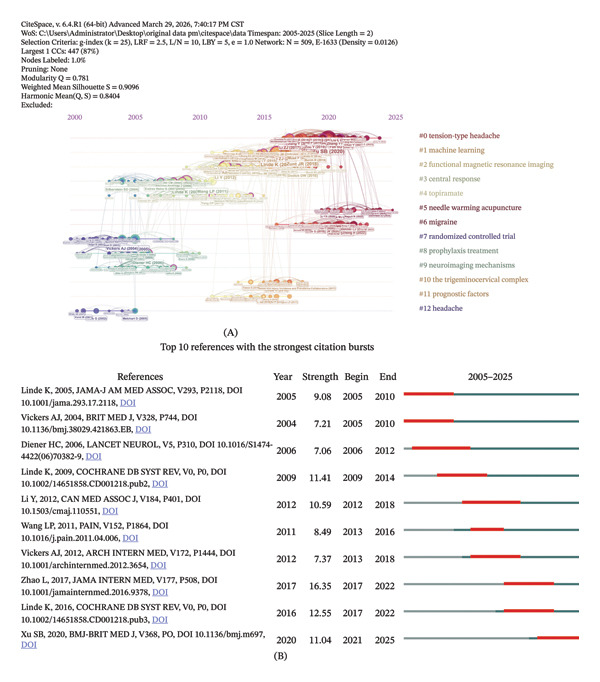
Reference clustering and citation burst analysis in acupuncture research on primary headaches. (A) Timeline clustering map of co‐cited references. (B) Top 10 references with the strongest citation bursts from 2005 to 2025.

The intellectual base of acupuncture research on PH is illustrated in Figure 7. Figure 7A presents the reference timeline clustering map, which identified several major knowledge domains, including TTH, randomized controlled trials, migraine, prophylaxis treatment, functional magnetic resonance imaging, neuroimaging mechanisms, and the trigeminocervical complex. The clustering structure demonstrated good reliability and consistency, with a modularity Q value of 0.781 and a weighted mean silhouette S value of 0.9096.

Figure 7B further shows the temporal evolution of influential references through citation burst analysis. Early bursts were dominated by landmark studies and evidence summaries published in *JAMA*, *BMJ*, and *Lancet Neurology*, whereas later bursts increasingly involved Cochrane reviews and rigorous randomized controlled trials. Notably, the study published in *JAMA Internal Medicine* by Zhao L et al. (2017) showed the strongest burst intensity (strength = 16.35) [20], followed by the meta‐analysis published in *Cochrane Database of Systematic Review*s by Linde K et al. (2016) [21] and the RCT published in *BMJ* by Xu SB et al. (2020) [22]. These findings indicate that high‐quality clinical trials and evidence syntheses have played central roles in shaping the intellectual base of acupuncture research on PH.

### 3.7. Thematic Structure and Topic Evolution

**FIGURE 8:**
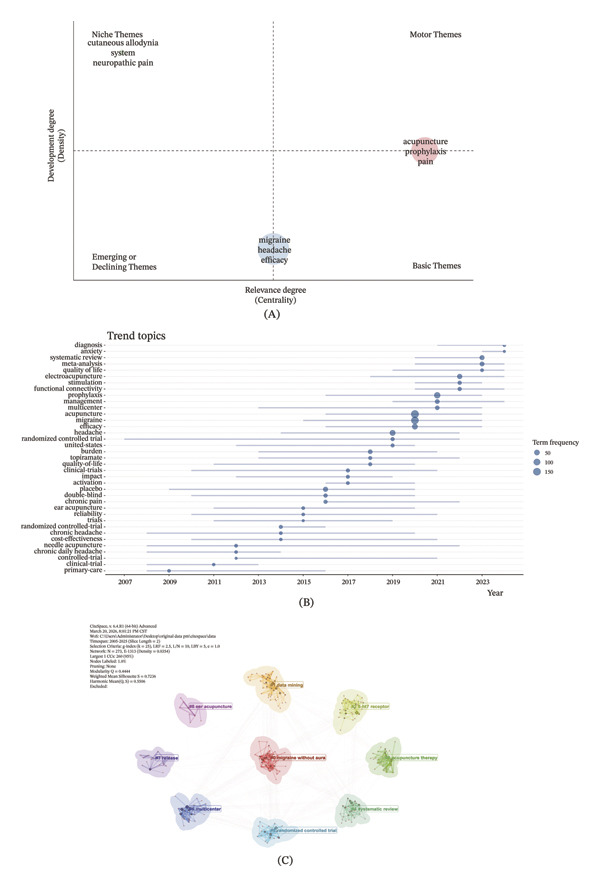
Thematic structure, trend topics, and keyword clustering in acupuncture research on primary headaches. (A) Thematic map based on author keywords. The horizontal axis indicates centrality, and the vertical axis indicates density. (B) Trend topic analysis showing the temporal evolution of major keywords from 2005 to 2025. (C) Keyword clustering map generated by CiteSpace.

The thematic structure and topic evolution of acupuncture research on PH are shown in Figure 8. Figure 8A presents the thematic map, which indicated that “acupuncture,” “prophylaxis,” and “pain” constituted the motor themes of the field, suggesting that these topics are both central and well developed. Meanwhile, “migraine,” “headache,” and “efficacy” appeared as basic themes, reflecting their continuing foundational roles in the literature. More specialized themes, such as cutaneous allodynia and neuropathic pain, were located in the niche‐theme quadrant, indicating relatively mature but less central subdomains.

Figure 8B shows the trend‐topic analysis, which further revealed a temporal shift in research focus. Early studies were more strongly associated with clinical trial design and healthcare application, including terms such as “clinical‐trial,” “controlled‐trial,” “needle acupuncture,” and “cost‐effectiveness.” In the middle period, terms related to placebo control, double‐blind design, quality of life, and multicenter studies became more prominent. In recent years, the field has increasingly focused on migraine‐specific management and mechanistic or evidence‐synthesis topics, including “electroacupuncture,” “functional connectivity,” “systematic review,” “meta‐analysis,” “anxiety,” and “diagnosis”. A further time overlay network is available in Figure S5.

Figure 8C presents the keyword clustering map, which identified several major thematic groups, including migraine without aura, acupuncture therapy, systematic review, randomized controlled trial, multicenter studies, and mechanism‐related topics such as the 5‐HT7 receptor. Taken together, these results suggest that the field has evolved from a stronger emphasis on clinical trial design and efficacy evaluation toward a more diversified structure involving evidence synthesis and emerging mechanistic exploration.

## 4. Discussion

### 4.1. Principal Findings of This Bibliometric Analysis

This bibliometric analysis provides an updated picture of acupuncture research on PH from 2005 to 2025. Several features of the field are particularly clear. First, the annual publication output increased overall, especially after 2018, indicating that interest in this topic has continued to expand rather than plateau. Second, the field is highly uneven in its geographical distribution. China contributed the largest share of publications and occupied the most central position in the current collaboration network, whereas the research output of most other countries remained relatively limited. Third, international collaboration was present at the country, institutional, and author levels, but it was mainly concentrated among a relatively small number of productive countries and established research groups. Fourth, the intellectual base of the field was dominated by randomized controlled trials, systematic reviews, and evidence syntheses, indicating that clinical efficacy evaluation has been the major driver of knowledge accumulation. Finally, thematic analyses showed that the field remains strongly centered on migraine, prophylaxis, efficacy, and headache‐related outcomes, whereas mechanistic themes such as functional connectivity, neuroimaging, and trigeminocervical pathways are visible but still secondary. Figure S6 further illustrates the relationships in institutions, authors, and keywords in the three‐field plot.

These findings suggest that acupuncture research on PH has moved well beyond isolated clinical observation and now has a recognizable academic structure. However, that structure is not evenly developed. The field is mature in some respects, particularly in migraine‐related clinical research and evidence synthesis, but is still narrow in others, including subtype coverage, broader international integration, and mechanistic depth. In other words, the field has become more productive and more organized, but not yet fully balanced.

A further point worth noting is that the knowledge base appears to be shifting in character. Earlier influential literature was more strongly connected with broad clinical medicine and landmark efficacy studies, whereas more recent cited journals and references increasingly cluster around neurology, neuroscience, and headache‐specific evidence frameworks. This pattern suggests that acupuncture research for PH is being discussed less as a peripheral complementary medicine topic and more as part of the broader scientific conversation on headache management.

### 4.2. Research Hotspots and Thematic Concentration in Acupuncture for PH

One of the clearest findings of the present study is the thematic concentration of the literature around migraine. Across keyword structure, thematic mapping, highly cited references, and trend analyses, migraine consistently occupied a central position. This pattern is not surprising, because migraine is both highly prevalent and clinically burdensome, and it has long served as a major target for nonpharmacological intervention studies [2, 23]. In contrast, TTH, although still represented in the knowledge base and clustering structure, occupied a clearly secondary position, while cluster headache and other less common PH subtypes were markedly underrepresented. Therefore, although the present study addressed PH as a broad category, the retrieved literature was in practice dominated by migraine and, to a lesser extent, TTH.

This imbalance should be interpreted as an important feature of the field rather than merely a limitation of the review framework. The predominance of migraine‐related literature likely reflects several factors, including greater disease burden [2], stronger patient demand for preventive and adjunctive treatment options [24], more established clinical trial traditions [25], and clearer integration with both pharmacological and nonpharmacological management frameworks [23, 26]. By contrast, cluster headache and some other PH disorders remain relatively underexplored in acupuncture research, possibly owing to lower prevalence, greater challenges in case recruitment, and a more limited evidence base overall [27]. This observation suggests that future work should pay greater attention to underrepresented PH subtypes if the field aims to claim broader relevance across PH disorders.

Thematic mapping and trend analysis also showed that the core hotspots of the field extend beyond disease labels alone. Terms such as acupuncture, prophylaxis, pain, efficacy, and headache‐related outcomes remained highly central, indicating that the literature has been strongly organized around treatment effectiveness and preventive management. In the middle stage of development, methodological terms such as placebo, double‐blind, multicenter, and quality of life gained prominence, suggesting maturation in trial design and increasing attention to study quality and patient‐centered outcomes. More recently, terms related to electroacupuncture, functional connectivity, systematic review, meta‐analysis, anxiety, and diagnosis have become more visible, indicating that the field is not only continuing to assess efficacy but is also expanding toward more refined intervention subtypes, evidence synthesis, and multidimensional characterization of patients.

At the same time, the thematic structure suggests that the field has not fully moved beyond an efficacy‐centered paradigm. Even when newer topics emerge, many of them still remain linked to intervention comparison, clinical endpoints, or evidence synthesis rather than fully established mechanistic frameworks. Thus, the current hotspot structure may be described as clinically mature but mechanistically incomplete. This is a notable feature of acupuncture research in PH and helps explain why the literature now appears methodologically richer than before, yet still only partially integrated with contemporary mechanistic headache science.

### 4.3. Collaboration Patterns and the Evolving Research Landscape

The collaboration patterns observed in this study suggest that the field is connected, but selectively so. At the country level, China clearly dominates in publication output and occupies the most central position in the collaboration network. The United States also functions as a major collaborative node, with Germany, Italy, the United Kingdom, and Switzerland playing visible roles. However, the predominance of SCP over MCP indicates that domestic research activity still accounts for much of the field’s output. For this reason, it would be inaccurate to describe the field as lacking collaboration, but it would be equally inaccurate to describe it as broadly integrated. It is better characterized as a field in which collaboration exists but is concentrated.

A similar pattern appears at the institutional level. The current collaboration network is centered on a small number of Chinese institutions, especially Chengdu University of Traditional Chinese Medicine, Beijing University of Chinese Medicine, China Academy of Chinese Medical Sciences, and Capital Medical University. This reflects the strong institutional foundation of acupuncture research in China, where traditional Chinese medicine universities and affiliated clinical systems provide a stable research base. At the same time, the burst analysis suggests that earlier periods of institutional prominence were more closely associated with Europe and North America. This contrast is useful because it shows not only where the field is centered now, but also how that center of visibility has shifted over time. This pattern may reflect differences between research productivity and citation accumulation. China currently contributes the largest volume of publications, especially in the recent period, whereas several highly cited studies from Europe and North America were published earlier and had more time to accumulate academic influence. In addition, some of these earlier studies were published in high‐impact general medical or neurology journals, which may have further increased their visibility and citation reach.

At the author level, the field is similarly structured around several recognizable clusters rather than a diffuse collaborative network. A few author groups account for much of the visible output, and some authors appear to function as links between subgroups. This kind of structure is common in developing research areas: it supports continuity and cumulative output, but it can also make the field heavily shaped by a limited number of long‐standing teams. In the present case, that may partly explain why some themes, especially migraine prophylaxis and efficacy evaluation, are comparatively mature, while other areas remain less developed.

The main implication here is not simply that “more collaboration” is needed. Rather, the field would benefit from a broader range of collaborations. At present, the strongest links are concentrated within a relatively narrow academic core. Future progress may depend less on increasing the number of publications alone and more on linking currently separated domains—for example, acupuncture‐focused clinical groups, headache specialists, neuroimaging researchers, pain scientists, and methodologists. Such connections would likely improve not only productivity, but also the explanatory and translational quality of the field.

### 4.4. Mechanistic Gaps and Future Research Directions

One of the clearest gaps revealed by this study is the mismatch between the maturity of clinical efficacy research and the relative immaturity of mechanistic research. The current literature contains a substantial body of work on randomized controlled trials, prophylaxis, efficacy, pain outcomes, and systematic reviews. By contrast, mechanism‐related themes are present but less central. This means that the field has become increasingly capable of asking whether acupuncture may benefit patients with PH but is still less capable of explaining how those effects are produced, whether they differ across headache phenotypes, and which biological pathways are most relevant.

Several reasons likely contribute to this imbalance. First, PH disorders are mechanistically heterogeneous. Migraine, TTH, and cluster headache do not share a single pathophysiological mechanism [28] and therefore cannot be expected to share a single explanatory framework for acupuncture response. Second, acupuncture itself is a complex intervention. Acupoint selection, stimulation mode, treatment intensity, frequency, course length, and sham design vary substantially across studies, which makes both mechanistic and clinical standardization research difficult [29]. Third, translational models remain limited. Compared with drug studies, acupuncture research still lacks widely accepted frameworks that can reliably connect animal work, clinical phenotypes, neuroimaging changes, and biomarker readouts. Finally, in a field still working to establish clinical credibility, it is unsurprising that efficacy and safety studies have developed faster than mechanistic investigations.

Even so, the present results point to several concrete directions rather than a vague need for “more mechanism research.” Neuroimaging and functional connectivity are already emerging within the field and provide one realistic entry point for future work. These approaches may help clarify whether acupuncture modulates large‐scale brain networks involved in pain processing, salience attribution, affective regulation, and descending pain control. Likewise, the appearance of themes related to the trigeminocervical complex suggests growing interest in brainstem–trigeminal mechanisms that are directly relevant to migraine biology [30]. Research that links acupuncture with trigeminovascular signaling, central sensitization, allodynia, and headache‐specific neurophysiological markers may therefore be especially valuable.

Another important direction is stratification. It is unlikely that all patients with PH respond to acupuncture in the same way. Clinical heterogeneity may be one reason why mechanistic findings remain fragmented. Future studies may be more informative if they distinguish patients by headache phenotype, chronicity, allodynia status, psychiatric comorbidity, autonomic features, or imaging‐defined subgroups. In parallel, biomarker‐based approaches—including neuroimaging indices, inflammatory signals, neuropeptides, and potentially CGRP‐related measures where appropriate—may help link symptom improvement to interpretable biological changes.

Methodologically, the field would benefit from designs that bridge clinical efficacy and mechanism more directly. Instead of separating clinical trials from mechanistic studies, future work could embed mechanistic endpoints within well‐controlled clinical studies. This would make it possible to evaluate not only whether acupuncture improves symptoms, but also whether specific neural, sensory, or molecular changes accompany clinical response. Such an approach would be more informative than parallel but disconnected streams of efficacy and mechanism research.

Overall, the field appears to be moving in this direction, but unevenly. The growing visibility of electroacupuncture, functional connectivity, anxiety, diagnosis, and evidence synthesis suggests that the research agenda is broadening. The next step is to make these strands converge into a more coherent translational framework.

### 4.5. Strengths and Limitations

This study has several strengths. First, compared with earlier broad bibliometric overviews, the present analysis applied a stricter manual screening process based on title, abstract, document type, publication year, language, and topic relevance. This allowed us to reduce the inclusion of records that were unrelated or only loosely related to acupuncture research on PH. Second, the study provides an updated overview covering publications through 2025 and integrates multiple analytical dimensions, including publication trends, country‐, institution‐, and author‐level collaboration, cited journal dynamics, reference bursts, thematic mapping, and trend‐topic analysis. Third, by combining productivity structure with intellectual‐base analysis and thematic evolution, the study not only describes who contributes to the field but also helps clarify how the field has developed and where its current imbalances lie.

Several previous bibliometric studies have examined acupuncture‐related research on headache, migraine, or TTH [15–17]. Our findings are broadly consistent with those reports in identifying migraine, clinical trials, and evidence synthesis as core components of the field. However, the present study extends that literature by applying stricter manual topic‐based screening, focusing explicitly on PH, and integrating country‐, institution‐, author‐, reference‐, and thematic‐level analyses up to 2025. In this sense, the value of the present work lies not in claiming to be the first bibliometric study in this broad area but in providing a more recent and more tightly screened map of the field while also highlighting the imbalance across PH subtypes and the gap between clinical evidence accumulation and mechanistic development.

Several limitations should also be acknowledged. First, the analysis was restricted to the WoSCC and to English‐language publications. Relevant studies indexed in other databases or published in other languages, particularly Chinese, may therefore have been missed. Second, bibliometric findings are inherently shaped by database indexing quality, citation behavior, and keyword standardization. Even with manual screening, some residual overlap with the broader pain and acupuncture literature may remain, particularly in peripheral thematic areas. Third, although the study focused on PH as a whole, the final literature corpus was dominated by migraine and, to a lesser extent, TTH, whereas cluster headache and other less‐studied PH subtypes were underrepresented. The conclusions of this analysis should therefore be interpreted primarily in relation to the more extensively studied portions of the PH spectrum. Finally, bibliometric analysis can identify structural patterns and research trends, but it cannot by itself establish clinical effectiveness or mechanistic validity. The present findings should therefore be understood as field‐level observations rather than direct evidence of therapeutic efficacy.

## 5. Conclusion

This bibliometric analysis shows that research on acupuncture for PH has grown steadily from 2005 to 2025 and has become increasingly structured, collaborative, and evidence‐oriented. The field is mainly centered on migraine, prophylaxis, efficacy, and headache‐related clinical outcomes, whereas cluster headache and other less‐studied PH subtypes remain underrepresented. Although clinical evidence has accumulated substantially, mechanistic and translational research remains comparatively underdeveloped. Future studies should place greater emphasis on underrepresented headache subtypes, broader interdisciplinary collaboration, and more rigorous mechanistic investigation.

## Author Contributions

Gui‐yu Liu, Jia‐yong‐ming Liu, and Wei Xie designed this study. Gui‐Yu Liu, Jia‐yong‐ming Liu, Han‐na Jin, Zi‐yan Chen, Xiao‐han Zhu, Wen‐jing Tong, Zi‐meng Lai, Jia‐geng Xie, and Qing‐yu Chang conducted bibliometric analysis and carried out data analysis. Han‐na Jin prepared the graphs and tables. Gui‐yu Liu and Jia‐yong‐ming Liu prepared the manuscript. Wen‐lin Yu and Wei Xie edited the manuscript.

## Funding

This study was supported by the National Natural Science Foundation of China (Grant Nos. 82374193 and 82574754), the National TCM Advantage Specialty Cultivation Unit, State Administration of Traditional Chinese Medicine of the People’s Republic of China (document no. [2024]90, granted to Huizhou Hospital of Guangzhou University of Chinese Medicine), the Clinical Collaboration Project between Traditional Chinese Medicine and Western Medicine in the Treatment of Major and Intractable Diseases (Migraine), State Administration of Traditional Chinese Medicine of the People’s Republic of China (granted to Nanfang Hospital, Southern Medical University), and the College Students Innovation and Entrepreneurship Training Program Project of Southern Medical University (Grant Nos. S202512121217 and 202512121408).

## Disclosure

All authors have read and approved it as the final manuscript.

## Ethics Statement

This study is not applicable for ICMJE registration number because it is not a clinical study.

## Conflicts of Interest

The authors declare no conflicts of interest.

## Supporting Information

Additional supporting information can be found online in the Supporting Information section.

## Supporting information


**Supporting Information 1** Figure S1. Supporting figure of Country analysis. (A) The cumulative growth of 10 most productive countries. (B) The international collaboration map, the depth of colour represents the number of articles published, and the thickness of lines represents the degree of cooperation.

**Supporting Information 2** Figure S2. Supporting institutional analysis. (A) The cumulative growth of 10 most productive institutions. (B) The overlap network among institutions.

**Supporting Information 3** Figure S3. Supporting author analysis. (A) The analysis of authors production based Lotka’s Law. (B) The cluster analysis of authors.

**Supporting Information 4** Figure S4. Supporting source analysis based on Bradford’s law.

**Supporting Information 5** Figure S5. Supporting keyword analysis.

**Supporting Information 6** Figure S6. Three‐field plot of institutions, authors, and keywords.

**Supporting Information 7** Table S1. The full records of 10 most cited publications.

## Data Availability

The data that support the findings of this study are available from the corresponding authors upon reasonable request.
