# Supplementary figures and images for: Mapping the Knowledge Landscape of Acupuncture for Primary Headaches: A Bibliometric Analysis From 2005 to 2025
Source: Pain Res Manag. 2026 Jul 31;2026:4922234. doi: 10.1155/prm/4922234 (PMC13426324; doi:10.1155/prm/4922234)

A

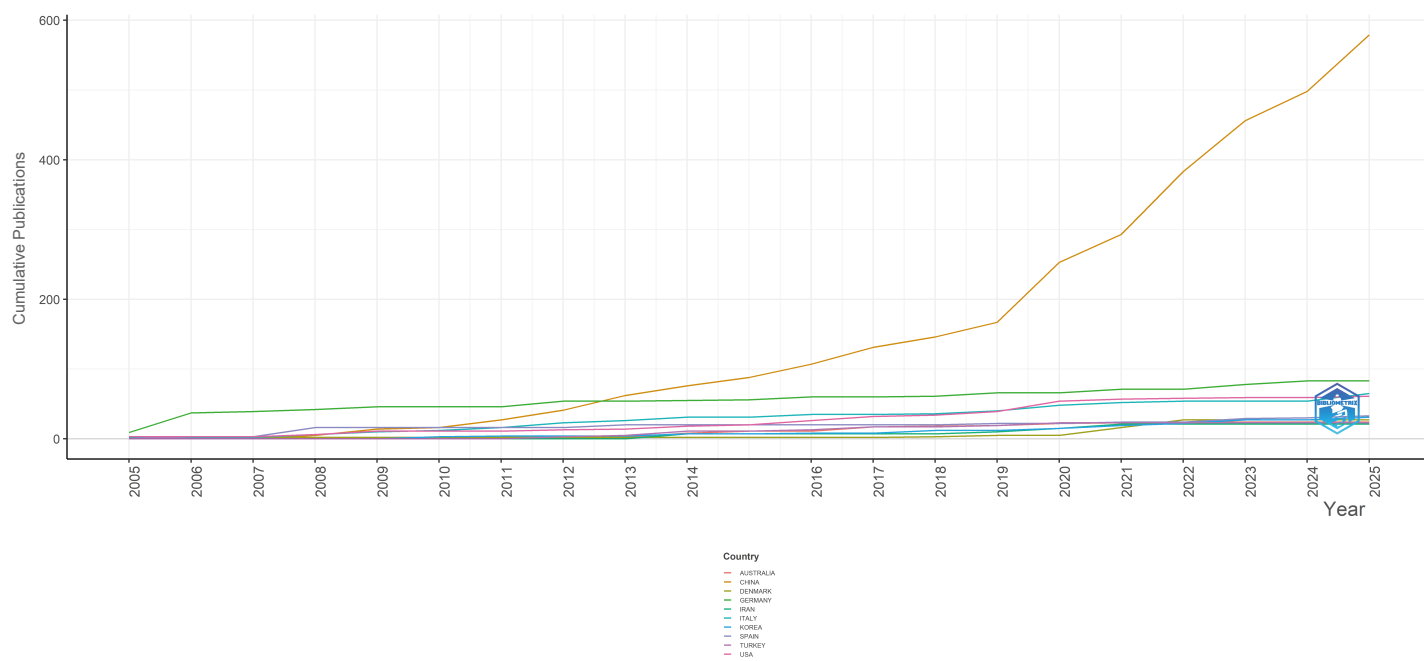

B

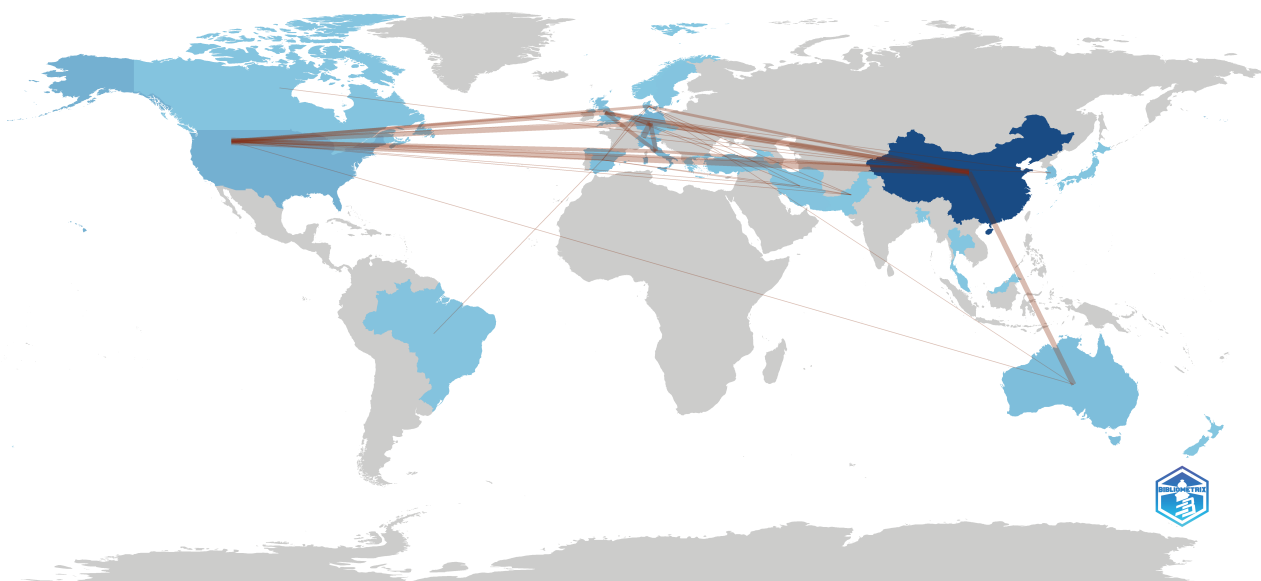

Supplement: Supplementary file 1 — Supporting Information 1 Figure S1. Supporting figure of Country analysis. (A) The cumulative growth of 10 most productive countries. (B) The international collaboration map, the depth of colour represents the number of articles published, and the thickness of lines represents the degree of cooperation. [file PRM-2026-4922234-s001.pdf]

## A Affiliations' Production over Time

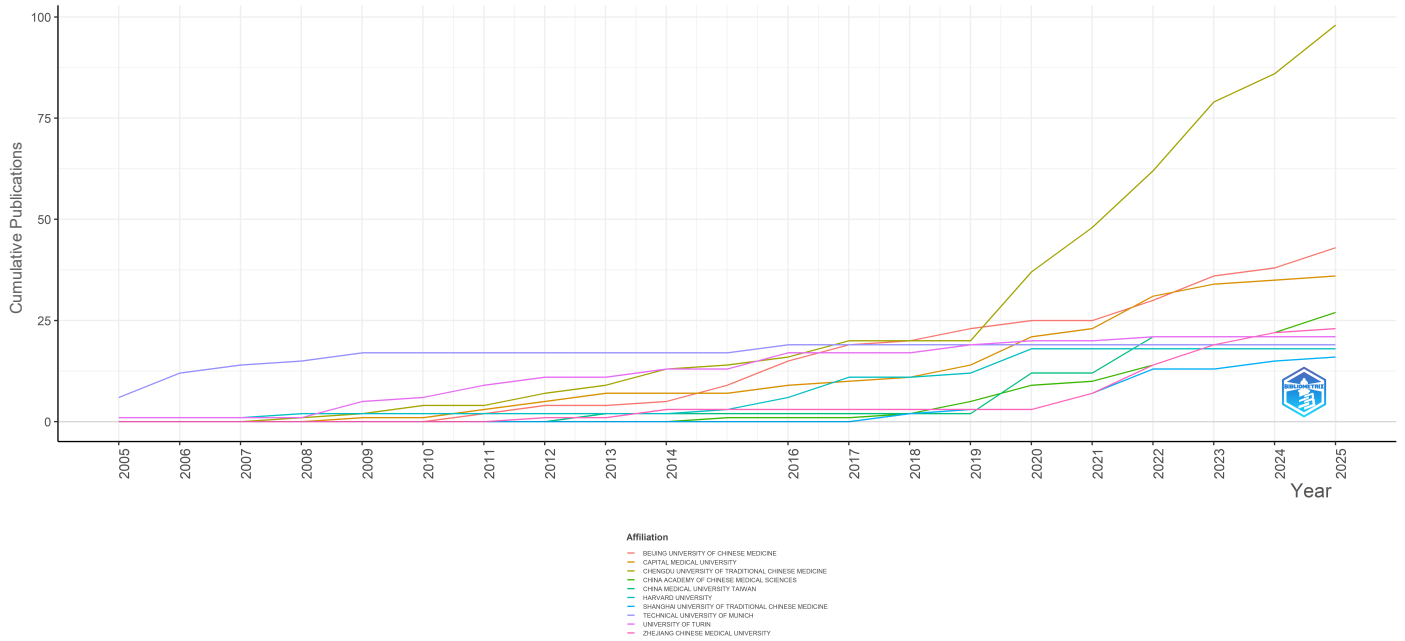

## B

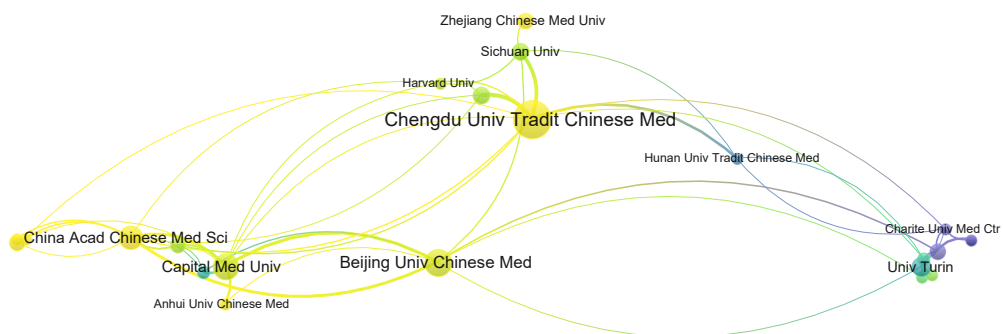

Supplement: Supplementary file 2 — Supporting Information 2 Figure S2. Supporting institutional analysis. (A) The cumulative growth of 10 most productive institutions. (B) The overlap network among institutions. [file PRM-2026-4922234-s002.pdf]

Core Sources by Bradford's Law

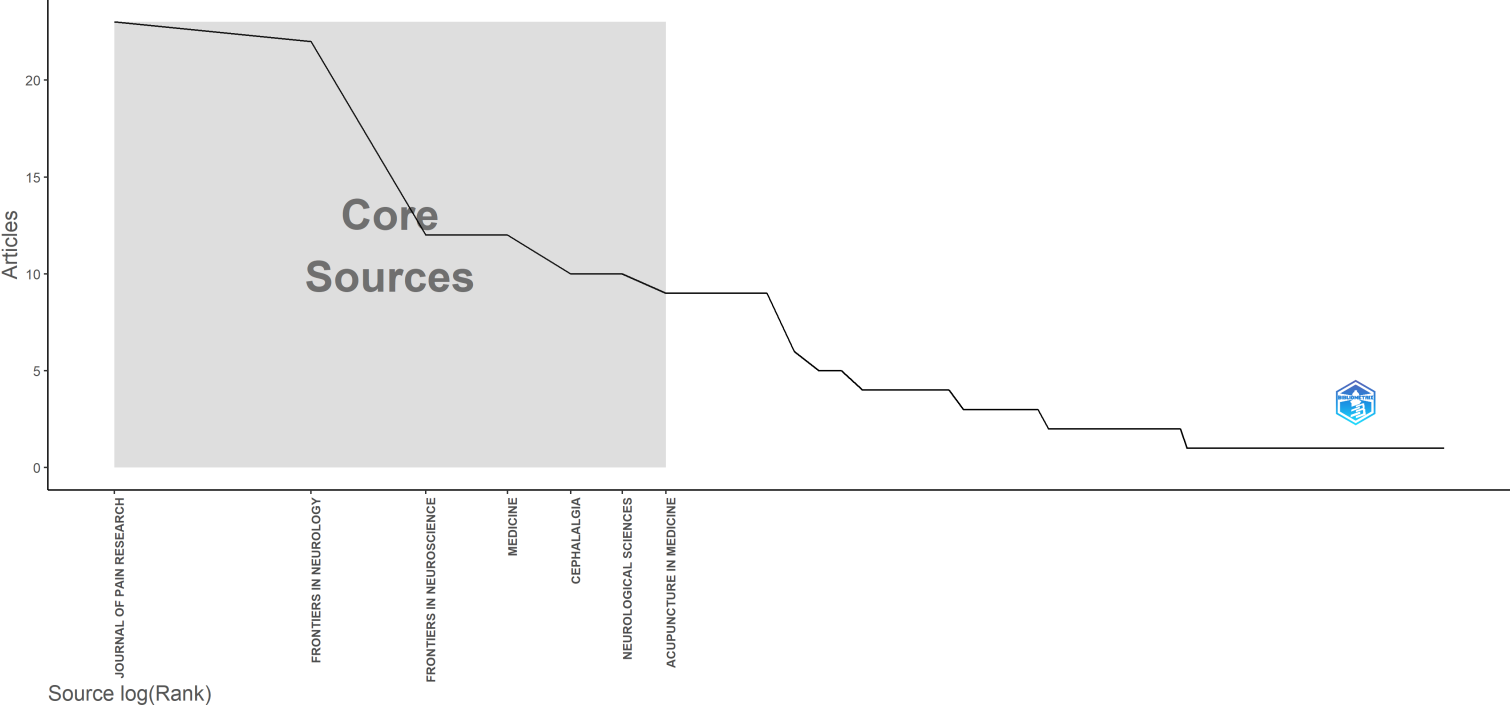

Supplement: Supplementary file 4 — Supporting Information 4 Figure S4. Supporting source analysis based on Bradford’s law. [file PRM-2026-4922234-s004.pdf]

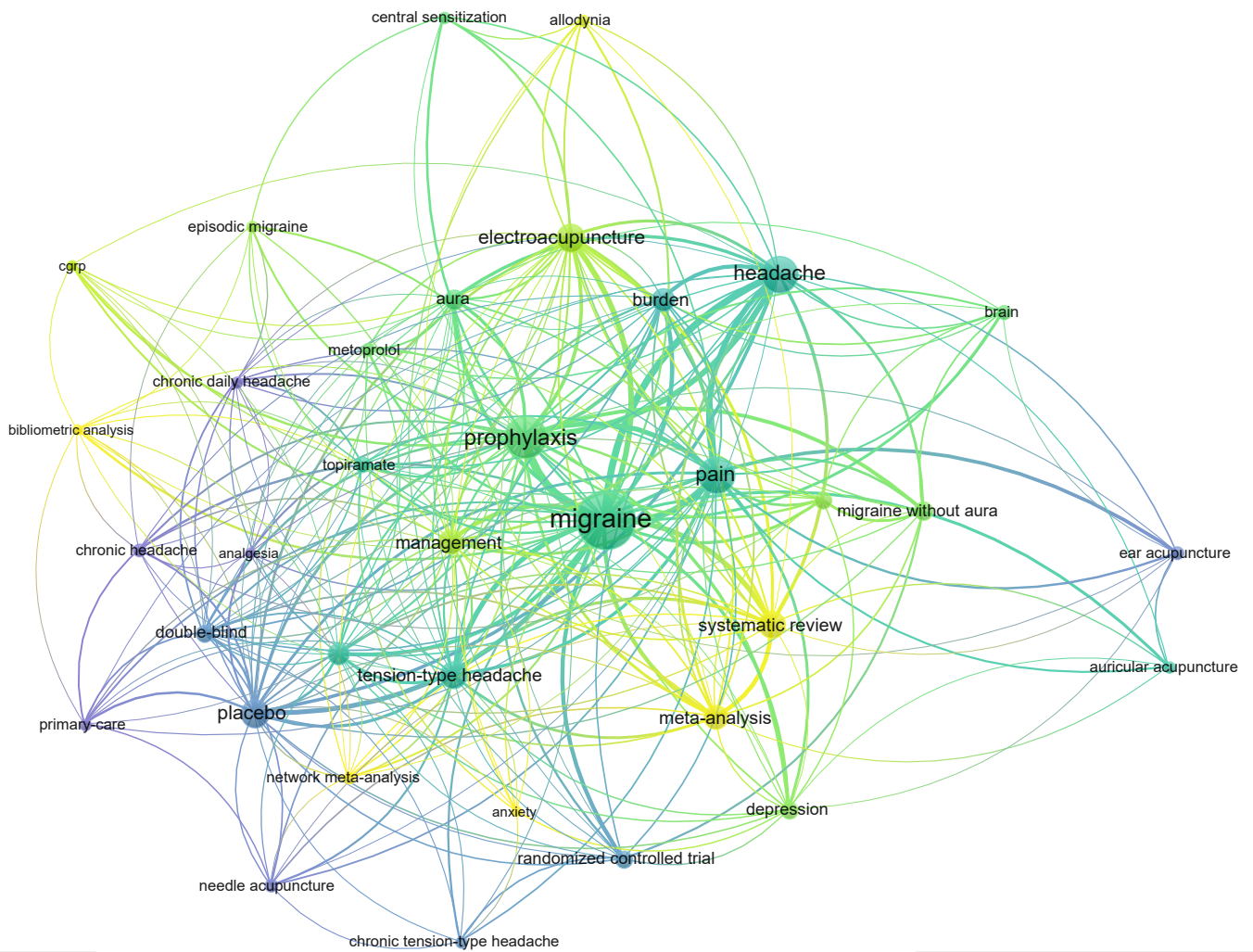

Supplement: Supplementary file 5 — Supporting Information 5 Figure S5. Supporting keyword analysis. [file PRM-2026-4922234-s005.pdf]

AU\_UN

AU

KW\_Merged

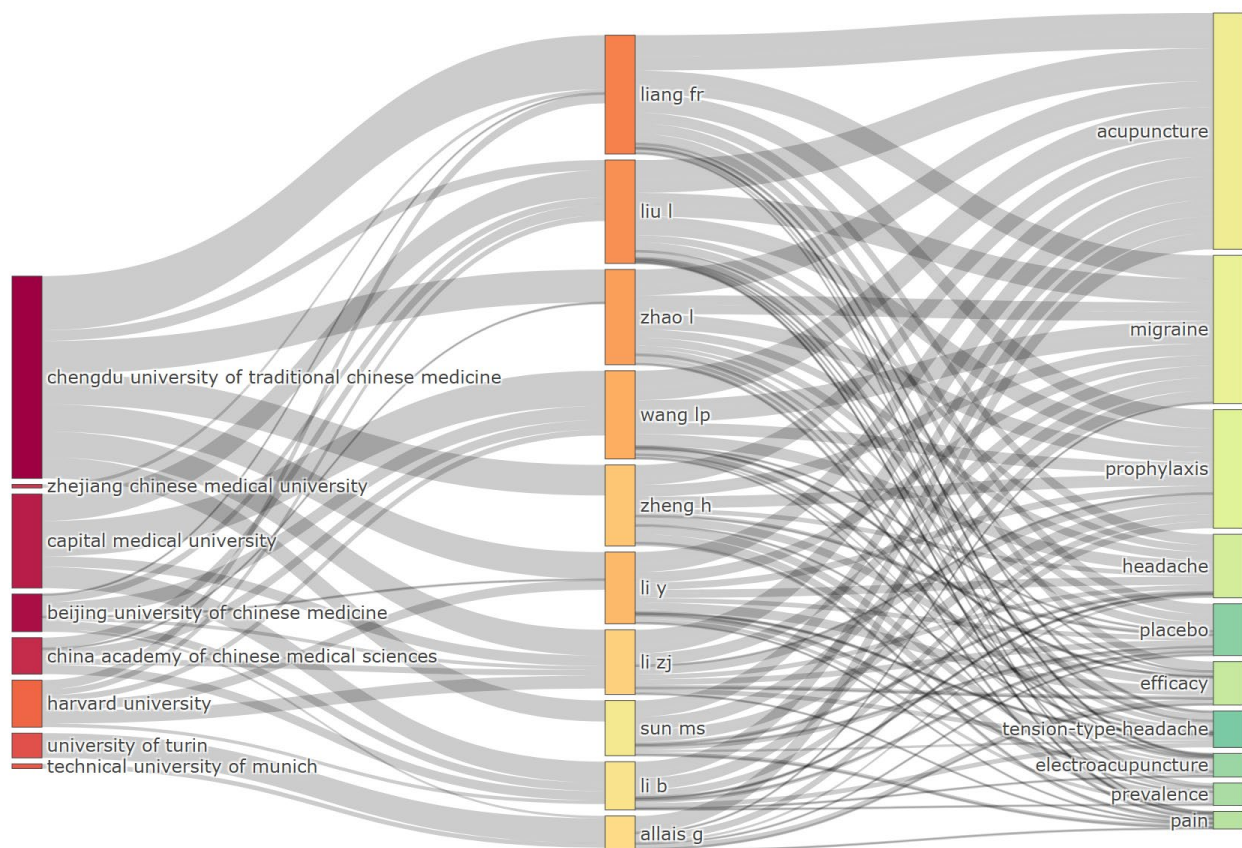

Supplement: Supplementary file 6 — Supporting Information 6 Figure S6. Three‐field plot of institutions, authors, and keywords. [file PRM-2026-4922234-s006.pdf]
