# Supplementary material for: Mapping the Knowledge Landscape of Acupuncture for Primary Headaches: A Bibliometric Analysis From 2005 to 2025
Source: Pain Res Manag. 2026 Jul 31;2026:4922234. doi: 10.1155/prm/4922234 (PMC13426324; doi:10.1155/prm/4922234)
Supplement: Supplementary file 3 — Supporting Information 3 Figure S3. Supporting author analysis. (A) The analysis of authors production based Lotka’s Law. (B) The cluster analysis of authors. [file PRM-2026-4922234-s003.pdf]

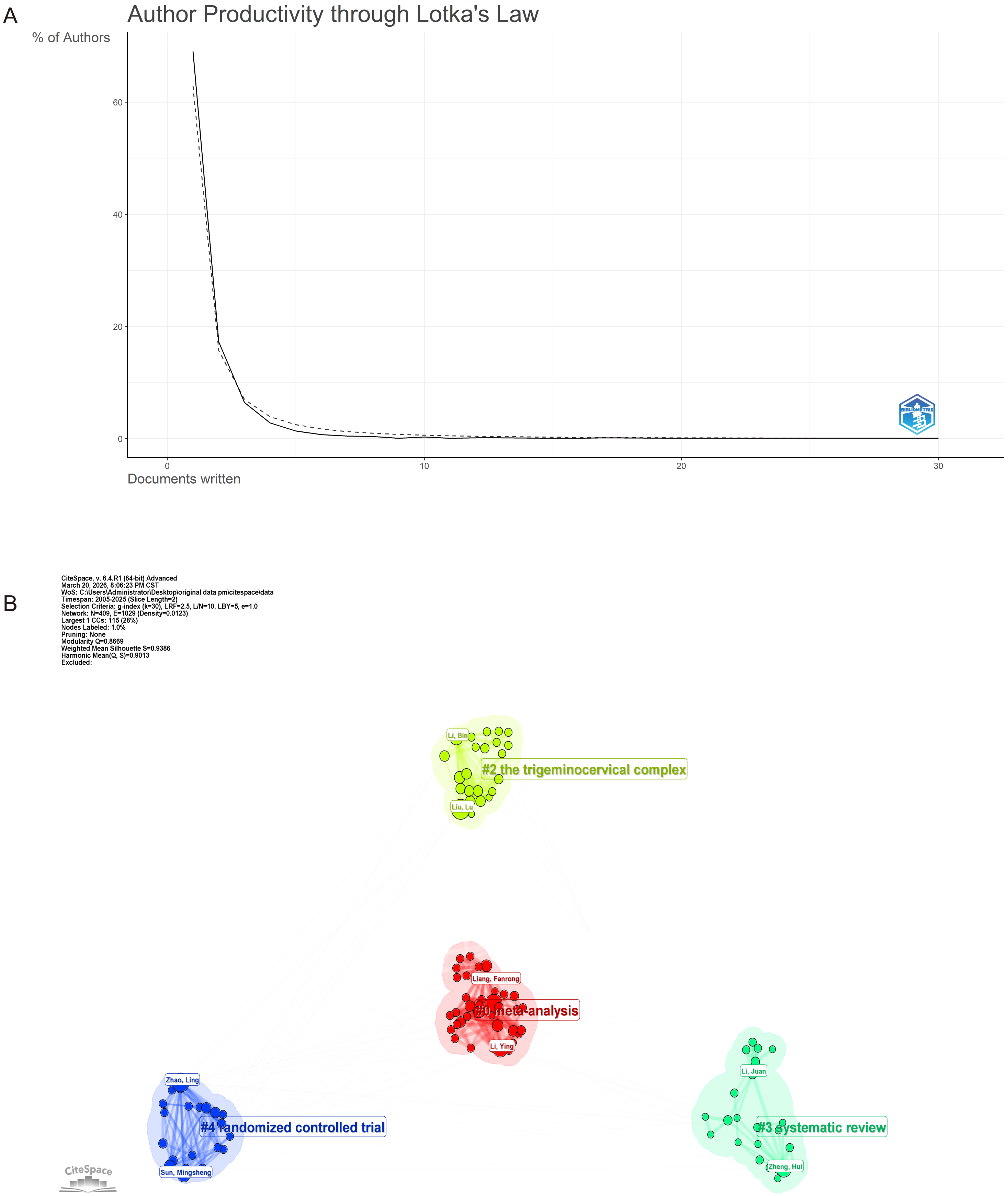
A
Author Productivity through Lotka's Law
% of Authors
60
40
20
0
0
10
20
30
Documents written
B
CiteSpace, v. 6.4.R1 (64-bit) Advanced
March 20, 2026, 8:06:23 PM CST
WoS: C:\Users\Administrator\Desktop\original data pm\citespace\data
Timespan: 2005-2025 (Slice Length=2)
Selection Criteria: g-index (k=30), LRF=2.5, L/N=10, LBY=5, e=1.0
Network: N=409, E=1029 (Density=0.0123)
Largest 1 CCs: 115 (28%)
Nodes Labeled: 1.0%
Pruning: None
Modularity Q=0.8669
Weighted Mean Silhouette S=0.9386
Harmonic Mean(Q, S)=0.9013
Excluded:
Li, Bin
#2 the trigeminocervical complex
Liu, Lu
Liang, Fanrong
#0 meta-analysis
Li, Ying
Li, Juan
Zhao, Ling
#4 randomized controlled trial
#3 systematic review
Sun, Mingsheng
Zheng, Hui
CiteSpace
